# Laparoscopic transperitoneal lateral adrenalectomy for malignant and potentially malignant adrenal tumours

**DOI:** 10.1186/s12893-015-0088-z

**Published:** 2015-08-28

**Authors:** Michał Pędziwiatr, Mateusz Wierdak, Michał Natkaniec, Maciej Matłok, Magdalena Białas, Piotr Major, Piotr Budzyński, Alicja Hubalewska-Dydejczyk, Andrzej Budzyński

**Affiliations:** 2nd Department of General Surgery, Jagiellonian University, Kopernika 21, 31-501 Kraków, Poland; Department of Physiology, Jagiellonian University, Grzegórzecka 16, 31-531 Kraków, Poland; Department of Pathology, Jagiellonian University, Grzegórzecka 16, 31-531 Kraków, Poland; Department of Endocrinology, Jagiellonian University, Kopernika 17, 31-531 Kraków, Poland

**Keywords:** Adrenocortical cancer, Pheochromocytoma, Adrenal metastasis, Laparoscopic adrenalectomy

## Abstract

**Background:**

Laparoscopic adrenalectomy is still controversial in cases where malignancy is suspected. However, many proponents of this technique argue that in the hands of an experienced surgeon, laparoscopy can be safely performed. The aim of this study is to present our own experience with the application of laparoscopic surgery for the treatment of malignant and potentially malignant adrenal tumours.

**Methods:**

Our analysis included 52 patients who underwent laparoscopic adrenalectomy in 2003–2014 due to a malignant or potentially malignant adrenal tumour. Inclusion criteria were primary adrenal malignancy, adrenal metastasis or pheochromocytoma with a PASS score greater than 6. We analyzed the conversion rate, intra- and postoperative complications, intraoperative blood loss and R0 resection rate. Survival was estimated using the Kaplan-Meier method.

**Results:**

Conversion was necessary in 5 (9.7 %) cases. Complications occurred in a total of 6 patients (11.5 %). R0 resection was achieved in 41 (78.8 %) patients and R1 resection in 9 (17.3 %) patients. In 2 (3.9 %) cases R2 resection was performed. The mean follow-up time was 32.9 months. Survival depended on the type of tumour and was comparable with survival after open adrenalectomy presented in other studies.

**Conclusions:**

We consider that laparoscopic surgery for adrenal malignancy can be an equal alternative to open surgery and in the hand of an experienced surgeon it guarantees the possibility of noninferiority. Additionally, starting a procedure with laparoscopy allows for minimally invasive evaluation of peritoneal cavity. The key element in surgery for any malignancy is not the surgical access itself but the proper technique.

## Background

Laparoscopic adrenalectomy is currently the gold standard for the surgical treatment of adrenal pathology [1, 2]. Initially, laparoscopy was only applied in the surgery of small adrenal tumours, the character of which indicated a benign lesion. Indications for laparoscopy have gradually broadened since it has been established that practically any tumour, including very large ones, can be successfully removed with this technique. It has obviously typical advantages for minimally invasive surgery: a lower complication rate, less postoperative pain, faster recovery, a shorter length of hospital stay and a better cosmetic effect [3, 4, 1, 5]. However, its use is still controversial in cases where malignancy is suspected. One of the concerns is that maintaining the proper oncological technique may be more difficult in comparison to open access, and thus there would be a higher risk of tumour capsule injury, which leads to intraoperative dissemination of cancer cells [6]. However, proponents of minimally invasive techniques argue that in the hands of an experienced surgeon, laparoscopy can be safely performed while preserving the principles of oncologic surgery, with results similar to those of open access [7]. The aim of this study is to present our own experience with the application of laparoscopic surgery in the treatment of malignant and potentially malignant adrenal tumours.

## Methods

Our analysis included patients who underwent laparoscopic adrenalectomy at our centre in the years 2003–2014 due to a malignant or potentially malignant adrenal tumour. The inclusion criterion was a postoperative pathological diagnosis confirming the character of the lesion: primary malignant tumour of the adrenal gland, pheochromocytoma with a high risk of postoperative malignant course or isolated adrenal metastasis after radical treatment of a primary lesion. We excluded from the study patients who were submitted to open surgery or patients with an inoperable tumour with distant metastases. Preoperative staging in all cases was comprised of computed tomography (CT), magnetic resonance imaging (MRI) or in selected cases a positron emission tomography (PET) scan. The criteria for potential malignancy in imaging studies included: invasion into adjacent organs or vessels, local or distant lymphadenopathy, increased and heterogeneous signal intensity on CT and on T2-weighted MRI, increased fludeoxyglucose uptake on PET and growth over time.

Prior to surgery a routine panel of laboratory tests were carried out to establish the hormonal activity of the tumour. The evaluation included plasma cortisol, urinary free cortisol, ACTH, DHEAS, 17-OH-progesterone, testosterone, plasma renin activity as well as aldosterone, urinary aldosterone, catecholamines and vanillylmandelic acid excretion. In the case of suspected pheochromocytoma, patients were preoperatively treated with alpha-blockers (doxazosin 20 mg/day) (additional beta-blockers in case of co-existing tachycardia) and intravenous volume expansion with crystalloids and colloids (2000 ml/day starting on the day before surgery).

We performed laparoscopy using the transperitoneal lateral approach. The same laparoscopic surgeon (AB) with extensive expertise in adrenal surgery (more than 370 laparoscopic adrenalectomies) performed all laparoscopic transperitoneal lateral adrenalectomies. The first attempt to use the laparoscopic approach in case of suspected malignancy was after completion of his learning curve (50 laparoscopic adrenalectomies). The principle during surgery was to avoid direct manipulating or applying pressure to the tumour to prevent perforation of the tumour capsule, or, in the case of pheochromocytomas, secretion of catecholamines. Any suspicion of damage to the tumour capsule was considered an indication for conversion. The adrenal gland with the tumour was resected with a margin of surrounding tissue and removed in a plastic laparoscopic bag. After surgery, patients were submitted to adjuvant therapy according to their primary oncological diagnosis. All the patients were subject to regular follow-up, which included clinical examination and imaging tests.

### Definitions

Primary tumours were defined as lesions originating in the adrenal gland. The potential risk of malignancy of the pheochromocytomas was evaluated with the Pheochromocytoma of Adrenal Gland Scaled Score (PASS); in postoperative pathological examination pheochromocytomas with a PASS score of 6 or higher were defined as potentially malignant [8]. Metastasis was defined as an adrenal tumour, discovered during follow-up diagnostic imaging, in patients with history of cancer in a different location, with a pathological diagnosis convergent with that of the later removed adrenal tumour.

The surgical procedure was described as R0 resection (macroscopically and microscopically radical), R1 resection (macroscopically radical, microscopically not radical – if the operative margin was smaller than 3 mm or when the pathologist was unable to definitively evaluate the radicality of the procedure in the report) or R2 resection (a procedure macroscopically and microscopically not radical or an intraoperative perforation of the tumour capsule).

The analysed measures were: conversion rate, intra- and postoperative complications, intraoperative blood loss and R0 resection rate. Survival was estimated using the Kaplan-Meier method. The study was conducted according to the Report of the ISPOR Task Force on Retrospective Databases [9]. All procedures were followed in accordance with the ethical standards of the responsible committee on human experimentation (institutional and national) and the Helsinki Declaration of 1975, as revised in 2008. The independent ethics committee of the Jagiellonian University, Krakow (KBET/45/B/2010) approved the study. Informed consent was obtained from all patients before surgery.

During the study period 495 patients were submitted to laparoscopy. In 60/495 (12.1 %) patients a significant suspicion of malignancy was established before surgery through clinical evaluation and diagnostic imaging. In this group, 30 (50 %) patients were operated due to suspected adrenal metastasis. The malignant character of the tumour was confirmed through pathological examination in 35 (58.3 %) out of the 60 patients. Moreover, in 17 (3.9 %) more patients, in whom the preoperative imaging did not clearly indicate malignancy, it was confirmed in the postoperative pathological examination. (Table 1). Further analysis included 52 patients with a postoperative diagnosis of malignancy or potential malignancy. This group included 23 women and 29 men. The mean age in the studied group was 57 years (with a range of 19–87 years). The demographic characteristics of this group are presented in Table 2.

**Table 1:** Preoperative and postoperative character of the removed adrenal tumours

Preoperative tumour with suspected malignancy *n* = 60	Postoperative benign tumour	*n* = 25 (41.7 %)
	Postoperative malignant tumour	*n* = 35 (58.3 %)
Preoperative tumour without suspicion of malignancy *n* = 435	Postoperative malignant tumour	*n* = 17 (3.9 %)
	Postoperative benign tumour	*n* = 418 (96.1 %)

**Table 2:** Demographic characteristics of the study group

Number of patients	52
Number of women	23 (44.2 %)
Number of men	29 (55.8 %)
Mean age	57 years (19–87 years)
Mean tumour size	58 mm (18–160 mm)
Left/right side	21/31

Fourteen (26.9 %) patients had a primary malignant adrenal tumour, 22 (42.3 %) adrenal metastasis and in 16 (30.8 %) pheochromocytoma with the malignant phenotype. Out of 14 primary malignant lesions, 3 (21.4 %) were hormonally active (2x glucocorticoid-secreting and 1 aldosterone-secreting). None of the metastases were hormonally active, whereas in 15/16 (93.7 %) cases of pheochromocytoma, catecholamines hypersecretion was confirmed preoperatively. Table 3 presents the pathological types of the removed lesions.

**Table 3:** Pathological types of the removed lesions

	Mean size	Histological type	*n* (%)	*n* (%)
Primary malignant adrenal tumour	74.9 mm (23 – 160 mm)	Adrenocortical cancer (ACC)	12 (23.1 %)	14 (26.9 %)
		Primitive neuroectodermal tumour (PNET)	1 (1.9 %)	
		Lymphoma	1 (1.9 %)	
Metastasis	46.9 mm (18–80 mm)	Renal cell carcinoma	11 (21.2 %)	22 (42.3 %)
		Non-small cell lung cancer	7 (13.5 %)	
		Colonic adenocarcinoma	2 (3.8 %)	
		Hepatocellular carcinoma	1 (1.9 %)	
		Sarcoma	1 (1.9 %)	
Malignant pheochromocytoma	59.8 mm (23–120 mm)	Malignant pheochromocytoma (PASS ≥ 6)	16 (30.8 %)	16 (30.8 %)
All	58 mm (18–160 mm)		52 (100 %)	52 (100 %)

## Results

In 47 (90.3 %) of the patients, the tumour was removed using the laparoscopic technique. Conversion was necessary in 5 (9.7 %) of the patients. The reasons for conversion are presented in Table 4.

**Table 4:** Reasons for conversion

Final diagnosis	Size (mm)	Reason of conversion
Pheochromocytoma	50	Infiltration to the back wall of the abdominal cavity
ACC	160	Suspicion of tumour capsule injury
Metastasis (renal cell cancer)	88	Adhesions after a previous surgery
ACC	120	Impossibility of safe laparoscopic dissection
Pheochromocytoma	120	uncontrolled bleeding

Mean blood loss was 220 ml (10–1500 ml). In 3 patients blood transfusions were necessary. Complications occurred in a total of 6 patients (11.5 %) and included hepatic parenchymal injury, damage to the splenic vein (not requiring splenectomy), aortic injury (during the open phase after conversion), pleural effusion and surgical site infection in 2 patients. None of the patients required reoperation. One female patient with multiple co-morbidities (ASA IV) with a pheochromocytoma that was additionally secreting corticosteroids required transfer to the intensive care unit on the day of surgery due to cardiopulmonary decompensation. This patient died on the 6^th^ postoperative day.

**Fig. 1:**
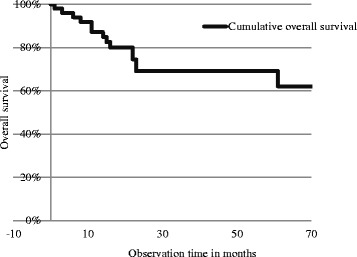
Kaplan-Meier survival curve for the entire group

**Fig. 2:**
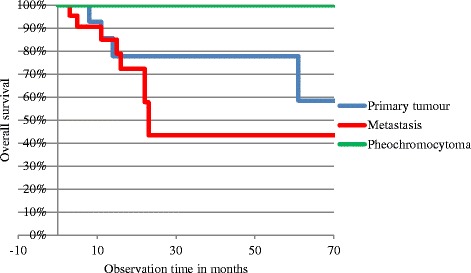
Kaplan-Meier survival curves for each tumor type

Pathologic results confirmed R0 resection in 41 (78.8 %) of the patients and R1 resection in 9 (17.3 %) of the patients. In 2 (3.9 %) of the patients R2 resection was performed. Postoperative mitotane therapy was applied in all patients with ACC and in 8 patients with metastasis, adjuvant chemotherapy was introduced according to the type of cancer. Patients with pheochromocytoma were not subject to adjuvant treatment. The mean follow-up time was 32.9 months (median: 24 months). One patient who died in the hospital was excluded from the analysis of follow-up time and long-term survival. The Kaplan-Meier survival curves for the entire group as well as for each tumour type are presented in Figs. 1 and 2.

## Discussion

According to most, if not all guidelines, any suspected adrenal malignancy is an indication for open adrenalectomy and this recommendation is very rarely questioned [10, 11]. Nowadays, most adrenalectomies worldwide are performed laparoscopically. Analyzing indications it becomes obvious that, at least in some cases, the abovementioned rule is silently and unnoticeably violated. Indeed, the true indication for the removal of incidentalomas results from concern that the early stages of cancer may have been overlooked [12]. All guidelines raise the issue of selection of cases where the risk of malignancy is high enough to justify adrenalectomy in hormonally silent tumours. As a natural consequence the firm adherence to this principle “always open surgery in any suspicion of malignant tumour” automatically implies that all incidentalomas should be operated classically. Meanwhile, looking at most series of minimally invasive adrenalectomy, at least half of all patients operated on for incidentalomas and internal contradiction using this approach is never questioned [13–15]. Thus, we believe that the opinion about open surgery for non-secreting tumours should be revised. In our series the risk of malignancy in unsuspected incidentalomas was 3.9 %. Similar data can be found elsewhere [15, 16]. This implies that removing every incidentaloma in this way would be unnecessary overtreatment in almost all cases.

Even though it may sound controversial, we believe that the same is true for cases with a higher risk of malignancy. The precise preoperative diagnostics of the potentially malignant character of pheochromocytomas remains an important challenge. It is generally accepted that in over 10 % of cases they can exhibit a malignant phenotype [17]. Only metastases observed at diagnosis or infiltration of surrounding organs observed at diagnosis are certain features of malignancy. This is, however, very rare. In most cases, they develop postoperatively in the follow-up period. Unfortunately, if there are no metastases, it is not possible to precisely evaluate the degree of malignancy based on biochemical and imaging tests [18, 19]. Pathological examination may also prove inconclusive. The most frequently employed scale for estimating the risk is PASS (Pheochromocytoma of the Adrenal Gland Scaled Score), proposed by Thompson in 2002 [20]. Though highly popular, its effectiveness for discovering malignant pheochromocytomas is highly debatable [21, 22]. Thompson has suggested that tumours with a PASS ≥ 6 were biologically more aggressive than tumours with a PASS <4. Meanwhile, Strong has observed that all patients in whom a progression of the disease was observed had tumours with a PASS over 6 [8]. This criterion was adapted for the purposes of our analysis. However, based on our observations, it appears that if there are no metastases during the operation, the risk of relapse is very low, since none of our patients relapsed. To some extent, this confirms the opinions that the PASS, despite being the most frequently used tool, does not allow for precisely predicting the postoperative course after the adrenalectomy for pheochromocytoma. An important conclusion arising from the analysis of our results is that laparoscopic adrenalectomy is a safe procedure in cases of pheochromocytoma considered as potentially malignant neoplasms. Additionally, if the pathological examination shows characteristics that may indicate a malignant phenotype, the procedure does not have a negative impact on the patient's outcomes, as compared to open surgery. Other authors also consider laparoscopic surgery a safe method, and recommend it as the method of choice, regardless of tumour size and the preoperative clinical picture, even though the malignant potential of pheochromocytoma is relatively high [23–25].

Nowadays, laparoscopic surgery has become a good alternative to open surgery in patients with isolated metastases. Despite certain controversy surrounding attempts at surgical treatment in patients with advanced stage cancer, it seems that, taking into account the advantages of minimally invasive techniques, laparoscopic adrenalectomy is perceived as an effective treatment method in chosen cases [26–28]. Research including relatively numerous groups of patients shows that such treatment enables prolonged disease free survival. In Moreno's study of 317 patients, the survival rate after one, two, three and five years was 80 %, 61 %, 42 % and 35 %, respectively [26]. Romero Arenas reported similar results (70 % survival after a year, 44 % after 3 years and 38 % after 5 years) [27]. Vazquez underlined one more important aspect – the treatment results depend also on the type of the primary tumour and thus may differ between study groups [29]. In our study, the survival rates for patients with metastases after a year, 2 years and 5 years were 82.4 %, 46.2 % and 40 %, respectively, which is similar to the results reported by other authors. The published papers comparing laparoscopic adrenalectomy with open surgery did not indicate any difference in survival rate between the methods, though they did note the obvious advantages of laparoscopy in early postoperative outcomes. All this contributes to a preference for laparoscopy in patients with metastases to the adrenal glands [30, 31, 26, 32–35, 29].

Our approach favours laparoscopy in all adrenal gland tumours, as it clearly emerges from this paper. In our series, in only 67.3 % (35/52) of the adrenal tumours with histologically confirmed malignant character was there significant suspicion of malignancy preoperatively. Furthermore, out of this group, 85.7 % (30/35) were patients with metastases. In other cases (17, 32.6 %) the character of the malignant or potentially malignant tumour was determined only postoperatively. Such a context calls into question the legitimacy of recommendations, which favour open techniques in these cases as nearly 1/3 of malignant tumours would be operated laparoscopically anyway. Some authors argue that in the case of primary adrenal malignancy, the preferred technique should be open surgery. They argue that local recurrence and peritoneal metastases occur sooner in patients who undergo laparoscopic adrenalectomy and positive resection margins are more frequently positive [36–39]. Others believe that the surgical approach does not have such a significant impact [7, 40, 41]. It seems that the role of the operative technique is the key question in this debate. In our opinion, laparoscopy differs only in that it involves a different access to the operated area. It allows for performing exactly the same operation meeting all the oncological principles (including in multi-organ resection and full lymphadenectomy) as long as it is performed by a skilled and experienced surgeon. We are certain that this aspect has more influence on the outcomes than the type of access itself. Moreover, in tumours located elsewhere (for instance, colon cancer), laparoscopy is an equal alternative to open operations, while giving the well-known advantages of reduced surgical trauma [42, 43]. It is even more so from a practical point of view, since a return to more frequent application of open surgery seems impossible, and often stands in contradiction to the expectations of both the patients and surgeons. It seems, however, reasonable that beginning every operation with laparoscopy, even in cases of larger and potentially malignant tumours, may be beneficial. It allows for a minimally invasive assessment of the entire abdominal cavity, searching for distant metastases and estimating the possibility of radical resection of the lesion without exposing the patient to large surgical trauma. Obviously the need for conversion arises whenever technical difficulties put into question the oncological quality of the procedure (dissecting the tumour, which could lead to damaging the tumour capsule, or doubts as to possible infiltration of neighbouring organs) [44, 5, 45, 11]. We also do not support laparoscopic surgery at all costs. Overall, conversion was necessary for 5 patients, out of which in 3 cases it was for “oncological reasons” (impossibility of dissecting the tumour, suspicion of capsule rupture). In the remaining 47 (90.3 %) patients, the procedure was carried out laparoscopically. The presented R0 resection rate of 78.8 % is a fully acceptable result, and certainly comparable, if not better than the results for open surgery [7, 36, 39]. This suggests that the laparoscopy allows for the achievement of satisfactory oncological outcomes. According to our data, the survival rate for patients with ACC after 1, 2, and 5 years was, respectively, 81.8 %, 70 % and 50 %. These results are slightly better than in other papers; however, they need to be interpreted with some caution, given the small study group size [46, 6]. Another issue is that we are the high-volume referral centre for adrenal pathologies and this is a proven factor for better outcomes [47, 48].

Our study is not free from limitations. It is a single centre retrospective study on a small sample, therefore defining confounders can be difficult and there is a potential for population bias. All procedures were performed by the same very experienced laparoscopic surgeon (>370 laparoscopic adrenalectomies), therefore the results probably cannot be simply transferred to all surgical departments.

## Conclusions

In summary, we consider that laparoscopic surgery for malignant or potentially malignant adrenal tumours is feasible. Safe laparoscopic surgery for any type of pheochromocytoma is a fact; especially that we are not able to distinguish a malignant from a benign tumour and even if it turns out to be potentially malignant, the long-term survival is very good. In patients with metastatic malignancy, adrenal metastasectomy seems to be a reasonable, less invasive solution allowing for comparable if not better results. Finally, the biggest concern remains primary adrenocortical cancer. Our results suggest that it is impossible to predict its occurrence in incidentalomas. In our opinion, if laparoscopy does not improve the outcomes, in the hand of an experienced surgeon it guarantees the possibility of noninferiority. Therefore, it can be recommended in most tumours. Additionally, beginning a procedure with laparoscopy allows for a precise, minimally invasive evaluation of the tumour stage. The key element in surgery for any suspected malignancy is not the surgical access itself but the proper technique in accordance with the principles of oncological surgery.

## Abbreviations

CT: Computed tomography
MRI: Magnetic resonance imaging
PET: Positron emission tomography scan
ACTH: Adrenocorticotropic hormone
DHEAS: Dehydroepiandrosterone sulphate
ACC: Adrenocortical cancer
PASS: Pheochromocytoma of Adrenal Gland Scaled Score
ASA: American Society of Anesthesiologists physical status
